# Metal oxide-based photocatalysts for the efficient degradation of organic pollutants for a sustainable environment: a review

**DOI:** 10.1039/d4na00517a

**Published:** 2024-08-22

**Authors:** Abdullah Al Miad, Shassatha Paul Saikat, Md. Kawcher Alam, Md. Sahadat Hossain, Newaz Mohammed Bahadur, Samina Ahmed

**Affiliations:** a Department of Applied Chemistry and Chemical Engineering, Noakhali Science and Technology University Noakhali Bangladesh; b Institute of Glass and Ceramic Research and Testing (IGCRT), Bangladesh Council of Scientific and Industrial Research (BCSIR) Dr Qudrat-i-Khuda Road, Dhanmondi Dhaka-1205 Bangladesh shanta_samina@yahoo.com

## Abstract

Photocatalytic degradation is a highly efficient technique for eliminating organic pollutants such as antibiotics, organic dyes, toluene, nitrobenzene, cyclohexane, and refinery oil from the environment. The effects of operating conditions, concentrations of contaminants and catalysts, and their impact on the rate of deterioration are the key focuses of this review. This method utilizes light-activated semiconductor catalysts to generate reactive oxygen species that break down contaminants. Modified photocatalysts, such as metal oxides, doped metal oxides, and composite materials, enhance the effectiveness of photocatalytic degradation by improving light absorption and charge separation. Furthermore, operational conditions such as pH, temperature, and light intensity also play a crucial role in enhancing the degradation process. The results indicated that both high pollutant and catalyst concentrations improve the degradation rate up to a threshold, beyond which no significant benefits are observed. The optimal operational conditions were found to significantly enhance photocatalytic efficiency, with a marked increase in degradation rates under ideal settings. Antibiotics and organic dyes generally follow intricate degradation pathways, resulting in the breakdown of these substances into smaller, less detrimental compounds. On the other hand, hydrocarbons such as toluene and cyclohexane, along with nitrobenzene, may necessitate many stages to achieve complete mineralization. Several factors that affect the efficiency of degradation are the characteristics of the photocatalyst, pollutant concentration, light intensity, and the existence of co-catalysts. This approach offers a sustainable alternative for minimizing the amount of organic pollutants present in the environment, contributing to cleaner air and water. Photocatalytic degradation hence holds tremendous potential for remediation of the environment.

## Abstract 2

Metal oxide-based photocatalyst can eliminate organic pollutants such as antibiotics, organic dyes, toluene, nitrobenzene, cyclohexane, and refinery oil from the environment.

## Graphical abstract

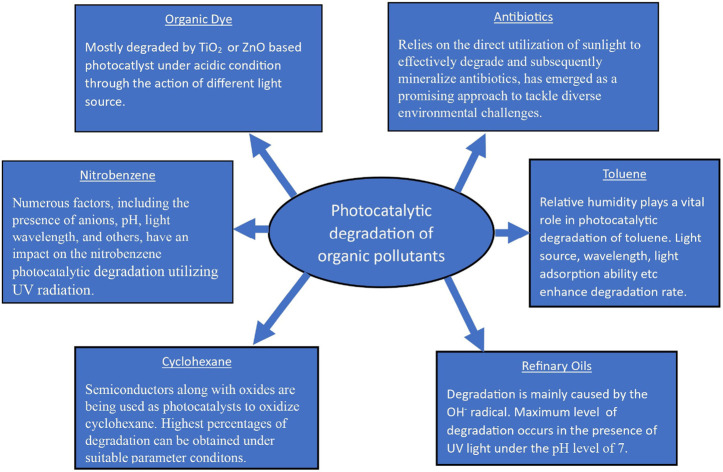

## Introduction

Urbanization and industrialization are cornerstones of modern civilization, underpinning significant advances in economic growth, technological innovation, and improved standards of living.^1^ These processes have facilitated the development of cities, expanded infrastructure, and increased industrial productivity, creating myriad opportunities for societal progress.^2,3^ However, the rapid pace of urbanization and industrialization has also ushered in substantial environmental challenges, particularly through the generation of wastewater that contains a diverse array of organic pollutants.^4–6^ These pollutants are frequently hazardous, presenting significant hazards to both the environment and public health, in contrast to conventional treatment procedures.^7–9^ Industrial operations are major contributors to wastewater pollution, as they produce effluents laden with complex organic chemicals.^10–12^ These chemicals are often by-products of various industrial processes and include a wide variety of substances such as antibiotics, organic dyes, nitrobenzene, cyclohexane, phenols, toluene, biphenyls, pesticides, fertilizers, hydrocarbons, plasticizing agents, detergents, oils, greases, proteins, and carbohydrates.^13–15^ The environmental impact of these pollutants is profound, as they can persist in the environment, bioaccumulate in wildlife, and enter human food chains, leading to chronic health issues and ecological damage.^16,17^ The complexity and resilience of these organic pollutants necessitate the development of advanced treatment technologies.^18,19^ Traditional biological treatment methods are often inadequate for fully degrading these pollutants due to their toxicity and chemical stability. In response to this challenge, Advanced Oxidation Processes (AOPs) have been developed and are increasingly being employed for the effective degradation of hazardous organic contaminants present in wastewater.^20–22^ AOPs are distinguished by the production of extremely reactive species, such as hydroxyl radicals, that can indiscriminately oxidize a broad spectrum of organic pollutants. This process converts the pollutants into less dangerous chemicals or fully mineralizes them into carbon dioxide (CO_2_) and water (H_2_O).^23^ Among the various AOPs, photocatalytic degradation stands out as a particularly effective method.^20^ Photocatalysis involves the use of semiconductor materials as catalysts to accelerate chemical reactions upon exposure to light. When semiconductor materials such as zinc oxide (ZnO), iron oxide (Fe_2_O_3_), titanium dioxide (TiO_2_), gallium phosphide (GaP), cadmium sulfide (CdS), and zinc sulfide (ZnS) are exposed to light, they generate electron–hole pairs that can generate reactive oxygen species.^24–26^ These reactive species possess the very capability of breaking down complex organic pollutants into less harmful, simpler molecules and fully mineralizing them.^27,28^ The advantages of photocatalysis are numerous and include low operational costs, the ability to accomplish full mineralization of contaminants without generating secondary pollution, and the capability to operate at ambient temperatures and pressures.^29^ Among the various photocatalysts, titanium dioxide (TiO_2_) is the most extensively studied and broadly applied because of its exceptional chemical and photochemical stability, cost-effectiveness, low toxicity, and high activity under ultraviolet (UV) light. TiO_2_, with its wide band gap of approximately 3.2 eV, can mineralize a broad spectrum of organic contaminants, including herbicides, dyes, pesticides, phenolic compounds, and pharmaceuticals like tetracycline and sulfamethazine.^30,31^ Nevertheless, the actual utilization of TiO_2_ is somewhat restricted due to its dependence on UV light, which comprises just a minor portion of the solar spectral region.^32^ To overcome this limitation, other semiconductor materials with broader light absorption properties are being explored. Tungsten trioxide (WO_3_) has emerged as a promising alternative due to its capability of absorbing visible light, making it more competent for photocatalytic oxidation of volatile organic pollutants under natural sunlight.^33,34^ Additionally, silver nanoparticles (AgNPs) have gained significant attention as photocatalysts due to their high photostability, environmental friendliness, and catalytic properties that are dependent on their shape and size.^35^ The effectiveness of photocatalytic systems in degrading organic pollutants is dependent on numerous operational parameters. These factors encompass the substrate concentration, photocatalyst quantity, pH of the solution, reaction medium temperature, light irradiation duration and intensity, photocatalyst surface area, dissolved oxygen content in the reaction medium, and the characteristics of both the photocatalyst and substrate.^29,36,37^ Furthermore, the doping of photocatalysts with metal and non-metal ions can enhance their photocatalytic activity by modifying their electronic properties and extending their light absorption range.^38^ It is important to optimize these parameters to maximize the degradation kinetics and overall efficiency of photocatalytic processes.^39^ For instance, the proportion of the substrate to the photocatalyst must be carefully balanced to ensure that there are enough reactive sites for pollutant molecules to adsorb and react.^37^ The pH of the solution can affect the charge and surface properties of the photocatalyst, influencing its interaction with pollutants. Temperature and light intensity also play significant roles in determining the rate of photocatalytic reactions, with higher temperatures and light intensities generally leading to increased reaction rates.^40–42^ In this review, we focused on the degradation of six specific types of organic pollutants: antibiotics, organic dyes, nitrobenzene, toluene, oil, and cyclohexane. These pollutants represent a broad spectrum of chemical structures and environmental impacts, making them ideal candidates for studying the effectiveness of various photocatalysts under different operational conditions. We will delve into the various reaction parameters that are critical to achieving maximum degradation of these pollutants using different photocatalysts. This comprehensive analysis aims to provide insights into the optimal conditions and catalyst selections for effective wastewater treatment, contributing to the mitigation of environmental pollution and the protection of aquatic ecosystems.

## Photocatalytic degradation of chemical pollutants (organic dyes and antibiotics)

Chemical pollutants refer to a large group of contaminants that arise from different sources, including pharmaceuticals,^43^ personal care items,^44^ pesticides,^45^ and other synthetic chemicals.^46^ Chemical pollutants, such as antibiotics and organic dyes, have significant adverse effects on the environment.^47^ Antibiotics, encompassing classes such as beta-lactams (*e.g.*, penicillins, cephalosporins), macrolides (*e.g.*, erythromycin), tetracyclines (*e.g.*, doxycycline), aminoglycosides (*e.g.*, gentamicin), quinolones (*e.g.*, ciprofloxacin), sulfonamides (*e.g.*, sulfamethoxazole), glycopeptides (*e.g.*, vancomycin), and oxazolidinones (*e.g.*, linezolid), are significant pharmaceutical pollutants.^48^ Organic dyes, including azo dyes (*e.g.*, methyl orange), anthraquinone dyes (*e.g.*, alizarin), phthalocyanine dyes (*e.g.*, copper phthalocyanine), triphenylmethane dyes (*e.g.*, malachite green), xanthene dyes (*e.g.*, fluorescein), and indigoid dyes (*e.g.*, indigo carmine), are prevalent industrial pollutants.^49^ Both types of pollutants are persistent in water bodies, posing substantial dangers to aquatic ecosystems and human health due to their toxicity, bioaccumulation potential, and the propagation of antibiotic-resistant bacteria.^50,51^ The persistence and toxicity of these chemical pollutants necessitate effective remediation strategies, such as photocatalytic degradation, which utilizes light-activated catalysts to break down these harmful substances into harmless by-products, ensuring cleaner water and healthier ecosystems.^52^

## Organic dyes

A significant group of synthetic organic molecules produced by a variety of industries, including the leather, plastic, food, paper, textile, and medicinal sectors, are known as dyes.^35,53^ Due to their frequent application in various manufacturing sectors, dyes are inevitably accidentally released into the environment, particularly into either surface water or groundwater, where they may pose serious dangers to environmental and biological systems.^54–57^ Over 700 000 tons of dyes are generated globally each year; 20% of these lost dyes reach the atmosphere and create pollution throughout processing or manufacturing, accounting for about 12% of the global total of dye generation. So the degradation of these organic dyes is necessary for maintaining the ecological balance.^58^ Organic dyes are very detrimental to aquatic ecosystems, even at low concentrations (less than 1 ppm). Thus, it is essential and required to remove organic dyes from effluents.^59^ The degradation mechanism of methylene blue dye is as follows.^60^

1 Photocatalyst + *hν* (photon) → Photocatalyst (e_cb_^−^ + h_vb_^+^)

2 e_cb_^−^ + O_2_ → ˙O_2_^−^

3 h_vb_^+^ + H_2_O → ˙OH + H^+^

4 h_vb_^+^ + OH^−^ → ˙OH

5 Methylene blue + ˙OH → Degradation products

6 Methylene blue + ˙O_2_^−^ → Degradation products

7 Degradation products + ˙OH/O_2_ → CO_2_ + H_2_O + Inorganic ions

Several metal oxides, such as ZnO, MgO, AgO, TiO_2_, Fe_2_O_3_, Mn_2_O_3_, CuO, and V_2_O_5_, are frequently employed as photocatalysts in wastewater treatment processes to degrade dyes.^61^ Zinc oxide (ZnO) is an oxidizing substance found in nature as the unusual mineral zincite. There have been attempts to use ZnO alongside other semiconductors for the photocatalytic degradation of an extensive variety of biological pollutants.^62^ ZnO-based photocatalysts work according to various parameter conditions. These parameters are mainly Ph, the initial concentration of dye or catalyst, the wavelength of the light & so on. The photocatalytic reaction rate at the outermost layer of the catalyst can be influenced by the initial concentration of the substrate. To prevent the dispersion of light and the concentration impact of the exposed photocatalyst surface, the ideal photocatalyst concentration ought to be unique for heterogeneous photocatalysis processes.^63^ Velmurugan *et al.* stated that the rate of degradation *k* dropped from 0.173 to 0.012 min^−1^ when the dye concentration was increased from 1 × 10^−4^ to 4 × 10^−4^ M.^64^ This is because many layers of adsorbed dye molecules have formed on the outermost layer of the catalyst, which prevents the photoreaction from occurring because there was not enough direct light interaction to produce hydroxyl radicals.^65^ The first amount of dye has a significant influence on the degradation efficiency of MB.^66^ Sobana *et al.* used ZnO that was manually combined with activated carbon (AC–ZnO) and solar irradiation to study the impact of initial Direct Blue 53 (DB53) concentration over the concentration range from 1 × 10^−4^ to 9 × 10^−4^ M.^67^ Its numerous functions make it extremely difficult to determine how the pH of a solution affects the efficacy of the dye photocatalytic degradation activity.^68^ Velmurugan *et al.* stated the impact of pH in the range of 3–11 upon the photocatalytic breakdown of Reactive Red 120 (RR 120) over ZnO during solar light irradiation.^64^ Photocatalytic breakdown of Reactive Orange 4 (RO4) and Black 5 (RB5) dyes occurs at various solution pH levels between 3 and 11.^69^ The pH, which regulates the adsorption of organic compounds on the outermost layer of the photocatalyst, serves as one of the most crucial factors influencing photocatalysis effectiveness.^70^ Electromagnetic relationships between the outermost layer of the photocatalyst and the substrate of interest can be employed to clarify how pH affects photocatalysis outcomes.^27^ Singh *et al.* stated that after exposing ZnO nanorods to UV radiation for 120 minutes, photodegradation activity levels were 7.169% and 47.63% for pH values of 4.5 and 10.5, correspondingly.^71^

**Fig. 1:**
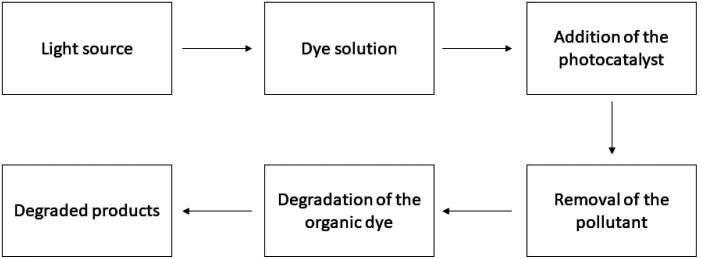
Working procedure of the photocatalyst for dye degradation.

Scientists' interest has been drawn more and more to supported TiO_2_ catalyst utilization over the past few years due to its prospective uses in the photocatalytic breakdown of organic contaminants such as organic dyes in air and water. Additionally, reports have it that when adsorbents are used to support TiO_2_, an ideal condition is created for the elimination or degradation of the compounds of interest.^72,73^ To enhance TiO_2_-based photocatalysts on organic dye in wastewater, several conditions were adjusted. These crucial elements, which included light intensity, TiO_2_ form and structure, target type, pH level and doping type, all had an impact on the photocatalysis method's effectiveness.^58^ If we want to discuss the parameters it is found that it is rather tough to comprehend how pH impacts the photodegradation process's efficacy.^29^ TiO_2_ exhibits amphoteric properties, allowing for the development of either a positive or negative charge on its outermost layer.^74^ Due to this, the adsorption of dye molecules over TiO_2_ surfaces may be affected by changes in pH.^75^ Bubacz *et al.* found that when pH is increased, so did the rate at which methylene blue was broken down photo-catalytically.^76^ On the other hand, Neppolian *et al.* showed that acidic conditions do not affect the degradation rate of the Reactive Blue 4 significantly enough.^77^ It has been found that organic dyes like Reactive Black 5 and Reactive Orange 4 degradation were enhanced in an acidic solution containing TiO_2_.^69^ Tanaka *et al.* discovered that at less acidic values, the positively charged TiO_2_ layer absorbed more Acid Orange 7, and greater breakdown was accomplished.^78^ A study has been conducted on the effects of pH on the adsorption as well as decolorization of Procion Red MX-5B (MX-5B) and Cationic Blue X-GRL (CBX). It was discovered that when the pH increased, MX-5B's adsorption was reduced.^62^ Another key parameter for dye degradation using a TiO_2_ catalyst is the dye amount or dye concentration. It has been found that the increased initial concentration of the dyes increases the degradation rate.^36,79^ This is because when the dye's initial concentrations rise, the dye molecules become deposited on the outermost layer of the catalyst and consume a sizable proportion of UV light instead of the TiO_2_ nanoparticles.^80,81^ Neppolian *et al.* investigated how the original dye concentration affected the percentage of degradation. With the best possible catalyst loading, they changed the starting concentrations of Reactive Yellow 17 (from 8.9 × 10^−4^ to 1.29 × 10^−3^ M), Reactive Red 2 (from 4.169 × 10^−4^ to 1.259 × 10^−3^ M), and Reactive Blue 4 (from 1.9 × 10^−4^ to 5.9 × 10^−4^ M).^77^ The dye degradation in a water-based solution utilizing a catalyst powder of TiO_2_ within a photocatalytic reactor is influenced by two additional parameters: the wavelength and intensity of the UV light irradiation source.^82^ Lower radiation wavelengths are thought to encourage the creation of electron holes, which would increase the catalyst's effectiveness.^83^ Ollis *et al.* said that at minimal light levels (0–20 mW cm^−2^), the rate would rise in an orderly manner as the intensity of light increased. The rate would rely on the square root of the light intensity at moderate light intensities (about 25 mW cm^−2^) but at intense light levels, the rate is independent of the light intensity.^29,84^ The degradation of Orange G was shown to be affected by light intensity in a range of 215 to 586 W cm^−2^. With a rise in light magnitude, Orange G's photolysis reaction rates climbed.^85^ Rao *et al.* stated that Acid Orange 7 (AO7) photocatalytically breaks down at a pace that is roughly 1.5 times faster in direct sunlight compared to that under synthetic UV radiation.^86^ Another significant operational parameter for the organic dye degradation is temperature range.^36^ The range of 40–50 °C was determined to be the ideal operating temperature range. Since desorption of the produced products happens more slowly at low temperatures than interface degradation as well as reactant adsorption, it restricts the reaction. Conversely, the limiting step becomes the dye's adsorption on TiO_2_ at an elevated temperature.^87^ A table has been added showing the photocatalytic degradation of organic pollutants (Table 1) and the process is illustrated in Fig. 1. The rate constant is lowered at elevated temperatures due to the organics' and hydrated oxygen's reduced adsorptive ability. Consequently, the ideal temperature often falls between 293 and 353 K.^108,109^

**Table 1:** Data for the photocatalytic degradation of organic dyes using various catalysts^a^

Pollutants	Pollutant conc. (mg L^−1^)	Catalyst	Catalyst conc. (mg L^−1^)	Operational conditions	Lamp, power (W)	Degradation percentage (%), time (min)	Ref.
MB	63.97	Mn-doped ZnO	—	Visible light; light intensity: 18.6 lux	Tungsten lamp, 500	50%, 10	88
IC	10	CA–CNT/TiO_2_–NH_2_	—	UV light (315–400 nm), pH = 2, temp = 80 °C	40	100%, 180	89
MB	30	CA–CNT/TiO_2_–NH_2_	—	UV light (315–40 nm), pH = 2, temp = 80 °C	40	80%, 300	89
RhB	6	Nanostructured TiO_2_	0.0001–0.0005	UV light, pH = around 7	—	93.8%, 190	90
AR57	30	TiO_2_	0.0005	UV light, pH = 7.18, temp = 400 °C	—	90.7%, 190	90
CR	75	ZnO	0.00016	Solar light, pH = 6	—	97%, 120	91
MB	50	Cu-doped ZnO (NPs)	—	Visible light	300	85%, 60	92
RhB	10	WO_3_/Ag_2_CO_3_	—	Visible light	Metal halide lamp, 70	99.7%, 8	93
MB	64	Undoped ZnO	—	Visible light; light intensity: 18.6 lux	Tungsten lamp, 500	50%, 30	67
MO	10	2% Al–2% Ni–ZnO	500	Visible light	Halogen lamp, 100	99%, 30	94
MB	—	Nano ZnO	—	UV light	Mercury vapor lamp, 8	97.64%, 120	95
MO	25	ZnO/Cu_2_O	—	UV light	Tungsten lamp, 200	73%, 180	96
AR27	—	Ce–ZnO	0.004	Solar light, degradation steadily increased up to pH = 12	—	90%, 60	79
MO	15	ZnO NFs	—	UV light	—	99.46%, 50	97
MO	—	Natural zeolite supported TiO_2_	0.0006–0.004	UV light, pH = 4	—	96.58%, 100	98
AB25	100	MgAl_2_O_4_ nanoparticles	—	UV, pH = 3	—	99.86%, 35	99
MB	50	ZnO NPs	—	UV/visible, —	—	100% under UV irradiation in 20 min, 91% under visible light in 60 min	100
RhB	10	TiO_2_/g-C_3_N_4_	—	Solar light, temp: 350 °C	Compact xenon lamp, 300	95%, 120	101
MO	10	[Zn(L)(H_2_O)]·H_2_O	—	UV light, —	High pressure Hg lamp, 300	83,8%, 120	102
MB	5	ZnO nanowires	—	UV light, —	High pressure Hg lamp, 50	96%, 120	103
IC	10	TiO_2_–NH_2_ NPs		UV light, pH = 2, temp: 80 °C	UV lamp, 40	100%, 180	89
RhB	0.001	Floral-like LaFeO_3_	—	Visible light, —	High pressure Hg lamp, 150	—, 720	104
RhB	23.4	ZnO	400	pH = 7	—	—, 180	105
RO16	20–60	TiO_2_	90	UV light, pH = 7.0, temp: 25 °C	Xenon lamp, —	87% after 20 min, 70% after 20 min, nearly 100% after 120 min	106
MO	—	PbBiO_2_Br	—	Visible light	Xe arc lamp, 300	95%, 60	107

a MB = Methylene Blue, IC = Indigo Carmine, RhB = Rhodamine B, AR57 = Acid Red 57, CR = Congo Red, MO = Methyl Orange, AR27 = Acid Red 27, AB25 = Acid Blue 25, RO16 = Reactive Orange 16.

## Antibiotics

Due to their extremely stable and non-biodegradable nature, antibiotics accumulate in the ecosystem as a result of overuse and uncontrolled environmental discharge.^110,111^ The release of diverse antimicrobial pollutants and their varied toxicity provide a significant challenge for researchers trying to find a solution.^112,113^ The excessive accumulation of antibiotics in natural environments has presented a significant peril to ecological systems.^114,115^ Unfortunately, traditional water treatment methods such as adsorption, filtration, and biodegradation are ineffective in effectively removing antibiotics due to their significant durability and limited biodegradability. Hence, the development of novel technologies is vital to ensure the efficient elimination of antibiotics.^116–119^ Due to its advantageous characteristics of cost-effectiveness, environmental sustainability, and high efficacy, heterogeneous photocatalysis has become a process of great promise for wastewater treatment, which relies on the direct utilization of sunlight to effectively degrade and subsequently mineralize organic pollutants, and has emerged as a promising approach to tackle diverse environmental challenges.^120–122^ Furthermore, it is crucial to provide an overview of frequently utilized photocatalytic nanomaterials and their specific use in breaking down popular antibiotics. This is necessary to validate their practical superiority and efficacy as catalysts for the process of photodegradation.^123–125^ The degradation mechanism of ciprofloxacin antibiotic in the presence of different photocatalysts is provided.^126^

8 Photocatalyst + *hν* (photon) → Photocatalyst (e_cb_^−^ + h_vb_^+^)

9 e_cb_^−^ + O_2_ → ˙O_2_^−^

10 h_vb_^+^ + H_2_O → ˙OH + H^+^

11 h_vb_^+^ + OH^−^ → ˙OH

12 Ciprofloxacin + ˙OH → Degradation products

13 Ciprofloxacin + ˙O_2_^−^ → Degradation products

14 Degradation products + ˙OH/O_2_ → CO_2_ + H_2_O + Inorganic ions

**Fig. 2:**
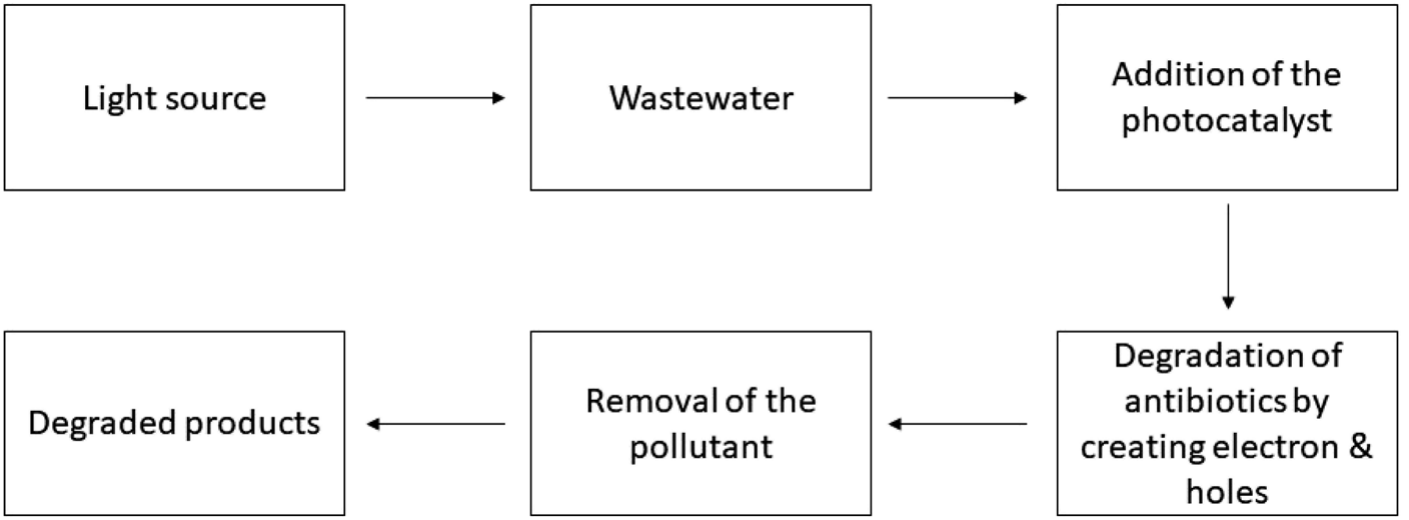
Working procedure of the photocatalysts for antibiotic degradation.

Yang *et al.* researched the degradation of ciprofloxacin using g-C_3_N_4_/TiO_2_ nanocomposites with the help of visible light irradiation utilizing a 300 W Xe visible lamp where the authors observed 88% of CIP degraded in 180 minutes.^127^ Verma explored the degradation of amoxicillin (AMX) by the utilization of TiO_2_ photocatalysis and sono-photocatalysis and achieved the highest degradation rate (80%) of AMX at a pH of 7.0 under UV irradiation at a power density of 672 W m^−2^.^128^ Zhang examined the mechanism and kinetics of photocatalytic degradation of tetracycline (TC) utilizing a supramolecular organic photocatalyst called three-dimensional network structure perylene diimide (3D-PDI).^129^ Fan *et al.* synthesized three different structures of bi-modified titanate nanomaterials (Bi-TNM) utilizing the hydrothermal technique and carefully adjusted variables to break down paracetamol (ACT). The study revealed that bi-titanate nanoribbons, when used at a concentration of 1 g L^−1^, had the most effective photocatalytic degradation capability, achieving a rate of 88%.^130^ The catalytic efficiency of NiS and NiS immobilized within the magnetite polypyrrole core/shell matrix (Fe_3_O_4_@PPY) was examined for the degradation of cephalexin. The study also examined the photocatalytic breakdown of cefalexin using the NiS-PPY-Fe_3_O_4_ photocatalyst, which was exposed to sunshine. The photocatalyst demonstrated a removal efficiency of over 80% over a 30 minute timeframe.^131^ Payan studied the creation of photocatalysts using Cu–TiO_2_@functionalized single-walled carbon nanotubes and found that sulfamethazine can be fully destroyed under solar irradiation within 300 minutes.^132^ R. Kumar *et al.* synthesized BN/CdAl_2_O_4_ composites and evaluated their photocatalytic ability to degrade cefoxitin sodium (CFT) antibiotic in an aqueous solution. The findings demonstrated that a nearly complete degradation of CFT, reaching approximately 100%, occurred within 240 minutes at a concentration of 15 mg L^−1^ and a pH of 7.^133^ A bismuth oxybromide (BiOBr) photocatalyst capped with PVP was produced by a solvothermal technique. The PVP-capped BiOBr exhibits a removal efficiency of 94% and 99.8% for the antibiotics ofloxacin (OFL) and norfloxacin (NOR) respectively, when exposed to visible light.^134^ Y. Gong prepared Z-scheme CdTe/TiO_2_ heterostructure photocatalysts decomposing 78% tetracycline hydrochloride (TC-H) within 30 min of irradiation under visible light.^135^ W. Wang examined the photocatalytic efficiency of BiVO_4_/TiO_2_/RGO composites for four tetracycline antibiotics. The BiVO_4_/TiO_2_/RGO photocatalyst demonstrated significant photocatalytic activity and compatibility, providing efficient separation of photo-generated carriers with oxidation capabilities and high reduction.^136^ N. Askari synthesized a novel heterojunction Z-scheme MnWO_4_/Bi_2_S_3_ using a hydrothermal technique to study the photocatalytic behavior of catalysts in the decomposition of metronidazole (MTZ) and cephalexin (CFX) under LED light exposure where a maximum degradation efficiency of 78.8% was achieved for CFX and 83.3% for MTZ.^137^ A. Mohammad *et al.* studied manufactured nanostructured photocatalysts composed of tin oxide (SnO_2_) and cerium oxide (CeO_2_). These photocatalysts were employed to degrade the antibiotic tetracycline hydrochloride (TC) under visible light. The most optimal outcome seen among the examined photocatalysts had a TC removal effectiveness of approximately 97% within a 120 minute timeframe under visible-light exposure.^138^ An investigation was conducted on the photocatalytic degradation of pharmaceutical micropollutants of Penicillin G (PG) in a photoreactor. The proficiency of the photocatalytic process was increased by the inclusion of persulfate sodium (PPS). The inclusion of PPS greatly enhanced the efficiency of the photolysis process, resulting in a considerable improvement of 72.72% compared to the traditional photocatalysis system, which achieved 56.71% efficiency.^139^ Bouyarmane synthesized TiO_2_-hydroxyapatite nanocomposites precipitating a re-dissolved natural phosphate mineral in ammonia using the concurrent gelation of titanium alkoxide. These nanocomposites were then subjected to degradation for drug testing in a solution under ultraviolet light. When utilizing 40TiHAp as a photocatalyst, ciprofloxacin and ofloxacin were destroyed through photodegradation in 15 minutes and 120 minutes, respectively.^140^ A simple solvothermal technique was employed to synthesize a novel Cu_3_P–ZSO–CN p–n–n heterojunction photocatalyst for the degradation of the antibiotic tetracycline (TC) under exposure to visible light. The degradation efficiency for TC was found to be 98.45%.^141^ M. Abdullah *et al.* synthesized ACT-X nanocomposites using activated carbon and TiO_2_ to enhance the inherent characteristics of TiO_2_, resulting in improved light absorption in the visible area. The ACT-4 photocatalyst has demonstrated the maximum level of photocatalytic degradation (99.6%) for the ceftriaxone (CEF) antibiotic.^142^ The very first 3D hierarchical ZnO/Bi_2_MoO_6_ heterojunctions were synthesized using an *in situ* solvothermal technique. These heterojunctions exhibited a remarkable efficiency of 100% in the photodegradation of the ofloxacin (OFL) antibiotic. This exceptional performance can be ascribed to their reduced electron–hole recombination rate and large surface area.^143^ A novel heterojunction photocatalyst (MoO_3_/g-C_3_N_4_) was synthesized using a straightforward hydrothermal calcination technique. The catalytic efficiency of this photocatalyst was assessed by measuring its ability to degrade tetracycline. The findings demonstrated that the 0D-2D MoO_3_/g-C_3_N_4_ Z-scheme heterojunction outperformed the original g-C_3_N_4_ and achieved an impressive 85.9% removal efficiency within 100 minutes when exposed to visible light.^144^ E. Gómez *et al.* fabricated highly efficient photocatalysts by using electrochemical deposition and thermal treatment. These photocatalysts, called nanostructured homojunction Bi_2_MoO_6_@Bi_2_MoO_6−*x*_, were able to effectively degrade and mineralize solutions containing various antibiotics (such as tetracycline, ciprofloxacin, and levofloxacin). After 180 minutes of radiation exposure, the photocatalysts achieved exceptionally high mineralization values (>95%) and near-complete degradation.^145^ P. Gholami *et al.* examined the photocatalytic efficacy of Zn–Co-layered double hydroxide (LDH) nanostructures containing charcoal (BC) in the breakdown of gemifloxacin (GMF), a representative pharmaceutical contaminant. The results indicate that 92.7% of GMF underwent degradation through photocatalysis in the presence of the Zn–Co-LDH catalyst. The effectiveness of BC-incorporated Zn–Co-LDH as a photocatalyst was greatly influenced by the concentration of the solute and the amount of photocatalyst used.^146^ Elegant Z-scheme composite hollow microspheres (CHMs) were made by sequentially controlling *in situ* hydrolysis and polymerization of WO_3_/g-C_3_N_4_. WO_3_/g-C_3_N_4_ CHMs are the most effective for photocatalytic degradation of CFS, with an 82% degradation efficiency after 2 hours of visible-light irradiation.^147^ Y. Sneha *et al.* conducted research on the properties of photocatalyst magnesium titanate (MgTiO_3_) in the presence of visible light, specifically focusing on its interaction with lomefloxacin. The study found that a concentration of 30 mg L^−1^ of catalyst was the most effective in breaking down 10 mg L^−1^ of lomefloxacin using 30 W LED irradiation for a duration of 150 minutes.^148^ The interaction between various surface facets of a semiconductor with suitable ratios can lead to improved performance in the degradation of photocatalytic processes. J. Wang *et al.* studied a material composed of bismuth called Bi_4_Ti_3_O_12_ and found that it showed improved degradation activity for tetracycline hydrochloride (TC-HCl) when exposed to irradiation.^149^ M. Shokri *et al.* investigated the degradation of cefazolin through exposure to immobilized and suspended TiO_2_ on a glass plate. A table has been added showing the photocatalytic degradation of antibiotics Table 2 and Fig. 2 shows the process. The findings indicate that the breakdown percentage of TiO_2_ suspension at favorable pH conditions (pH 5) is 96.47% after 60 minutes of irradiation.^150^

**Table 2:** Data for the photocatalytic degradation of antibiotics using various catalysts

Antibiotics^a^	Antibiotics conc. (mg L^−1^)	Catalysts	Catalyst conc. (mg L^−1^)	Operational conditions	Lamp, power (W)	Degradation percentage (%), time (min)	Ref.
CIP	10	g-C_3_N_4_/TiO_2_	375	Visible light	Xe, 300	88, 180	127
AMX	30	TiO_2_	450	UV light, pH 7	672	80, 270	128
TC	50	3D-PDI	25	Visible light, pH 5	—	80, 150	129
APAP	0.7	BiTNMs	1000	Visible light, pH 7	500	88, 180	130
CFX	50	NiS-PPY-Fe_3_O_4_	4000	UV light, pH 5.5	Hg, 75	80, 30	131
SMZ	30	Cu–TiO_2_@functionalized SWCNT	900	UV-vis light, pH 7	—	100, 300	132
CFT	15	BN/CdAl_2_O_4_	330	UV light, pH 7	108	100, 240	133
OFL	5	PVP capped BiOBr	10	Visible light	15	94, 240	134
NOR	5	PVP capped BiOBr	10	Visible light, pH 7.54	15	99.8, 240	134
TC-H	20	CdTe/TiO_2_	600	Visible light	300	78, 30	135
TC	0.01	BiVO_4_/TiO_2_/RGO	—	Visible light, pH 3	Xe, 1000	96.2, 120	136
CTC	0.01	BiVO_4_/TiO_2_/RGO	—	Visible light, pH 3	Xe, 1000	97.5, 120	136
OTC	0.01	BiVO_4_/TiO_2_/RGO	—	Visible light, pH 3	Xe, 1000	98.7, 120	136
DXC	0.01	BiVO_4_/TiO_2_/RGO	—	Visible light, pH 3	Xe, 1000	99.6, 120	136
CFX	20	MnWO_4_/Bi_2_S_3_	1200	Visible light	Xe, 1000	78.8, 180	137
MTZ	20	MnWO_4_/Bi_2_S_3_	1200	Visible light	Xe, 150	83.3, 180	137
TC	10	SnO_2_/CeO_2_	200	Visible light, pH 9–10	500	97, 120	138
PG	5	ZnO	800	UV light, pH 6.8	24	72.72, 150	139
CIP	20	40TiHAp	2000	UV light, pH 6.1	125	100, 15	140
OFL	20	40TiHAp	2000	UV light, pH 6.1	125	100, 120	140
TC	10	Cu_3_P/ZnSnO_3_/g-C_3_N_4_	500	Visible light	Xe, 500	98.45, 60	141
CEF	100	ACT-4	844	Visible light	LED bulb, 50	99.6, 260	142
OFL	10	ZnO/Bi_2_MoO_6_	250	Visible light, pH 7.54	Daylight lamp, 15	100, 240	143
TC	10	MoO_3_/g-C_3_N_4_	500	Visible light	—	85, 9100	144
TC, CPX, and/or LFC solution	60	Bi_2_MoO_6_@Bi_2_MoO_6−*x*_	300	Visible light, pH 7	LEDs, 6.2	>95, 180	145
GMF	15–35	Zn–Co-LDH@BC	750	UV light, pH 5.5	10	92.7, 130	146
CFS	500	WO_3_/g-C_3_N_4_	25	UV light	Xe, 300	82, 120	147
Lomefloxacin	10	MgTiO_3_	30	UV light, pH 7	LED light, 30	83, 150	148
TC–HCl	20	Bi_4_Ti_3_O_12_	10	UV light	Xe, 300	75.5, 150	149
Cefazolin	20	TiO_2_	400	UV light, pH 5	15	96.47, 60	150
SMX	—	TiO_2_	0.002	UV light	Xenon, 1500	88, 360	151
SMX	10	TiO_2_	250	UV light, pH 4.1–5.4	UV, 9	Close to 100, 120	152
OA	20	Titanium Degussa P-25	0.001	pH 7.5	Black light lamp	90, 30	153
Norfloxacin	—	C–TiO_2_	0.0002	Visible light	—	78, 70	154
TC	40	Metal ion@TiO_2_/HNTs		Visible light	Xenon, 500	76.54, —	155
Chloramphenicol	50	TiO_2_	0.001	UV light	Osram Dulux, 9	90, 90	156
SMX	100	TiO_2_	0.0005	UV light	Xenon lamp	80, 360	157

a CIP = ciprofloxacin, AMX = amoxicillin, TC = tetracycline, APAP = acetaminophen, CFX = cephalexin, SMZ = sulfamethoxazole, CFT = cefoxitin sodium, OFL = ofloxacin, NOR = norfloxacin, CTC = chlorotetracycline, OTC = oxytetracycline, DXC = doxycycline, MTZ = metronidazole, PG = penicillin G, CEF = cefixime, GMF = gemifloxacin, CFS = ceftazidime, SMX = sulfamethoxazole, OA = oxolinic acid.

## Other industrial pollutants (toluene, nitrobenzene, cyclohexane, and refinery oil)

Industrial chemical pollutants are a subgroup of chemical pollutants specifically connected with industrial operations.^158^ They encompass a wide spectrum of chemicals used or produced in manufacturing, refining, and other industrial processes.^159^ Industrial chemical pollutants, including toluene, cyclohexane, nitrobenzene, and refinery oil, pose significant environmental threats due to their widespread use and high toxicity.^160,161^ Toluene, an industrial solvent, pollutes air, water, and soil, causing harm to aquatic organisms and long-term environmental damage.^162^ Cyclohexane, used in chemical production, contributes to air and water pollution, affecting aquatic life.^163^ Nitrobenzene, a dye and pharmaceutical precursor, contaminates soil and water, posing toxic and carcinogenic risks.^164^ Refinery oil, a byproduct of petroleum refining, causes extensive damage through spills and leaks, affecting marine and terrestrial ecosystems.^165^ Photocatalytic degradation is crucial for mitigating these pollutants, as it offers an efficient, eco-friendly method to break down these toxic substances, preventing their persistence in the environment and safeguarding both ecosystems and human health.^166^

### Toluene

As one of the pollutants that pose a risk to human health and the ecosystem, toluene has been classified as a priority pollutant; for this reason, emission management is required.^167,168^ Owing to the serious issues that toluene causes, various methods for toluene abatement have been developed.^169^ The rapid growth in industrialization and urbanization has played a notable role in the emergence of severe environmental issues.^170,171^ Toluene, a volatile organic molecule, can induce skin inflammation, respiratory ailments, chronic and acute intoxication, neurotoxicity, and reproductive toxicity.^172–176^ Therefore, it is necessary to enhance the efficacy of eliminating indoor toluene vapors. Methods to counteract atmospheric pollution can be classified as either chemical or physical approaches.^177,178^ Physical approaches include adsorption, the process of condensation, and separating membranes. Chemical approaches encompass combustion, low-temperature plasma, biological, and photocatalytic treatments.^179,180^ Photocatalysis is regarded as a very promising option for environmental cleaning among these techniques. Photocatalytic technologies provide the benefits of being non-toxic and cost-effective, requiring gentle reaction conditions, and producing no secondary pollutants.^136,181^ Almost all the hydrocarbon degrades *via* the following mechanism.^182,183^

15 Photocatalyst + *hν* (photon) → Photocatalyst (e_cb_^−^ + h_vb_^+^)

16 e_cb_^−^ + O_2_ → ˙O_2_^−^

17 h_vb_^+^ + H_2_O → ˙OH + H^+^

18 h_vb_^+^ + OH^−^ → ˙OH

19 Toluene + ˙OH → Hydroxylated intermediates

20 Hydroxylated intermediates + ˙OH → Degradation products

21 Degradation products + ˙OH/O_2_ → CO_2_ + H_2_O + Inorganic ions

**Fig. 3:**
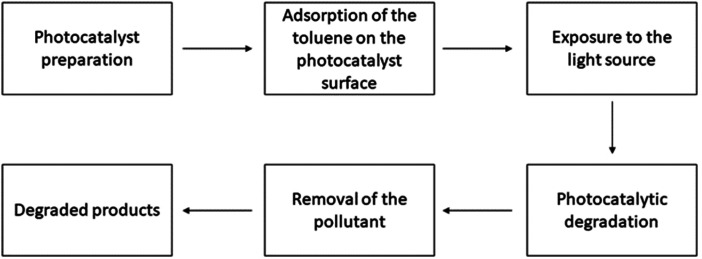
Working procedure of the photocatalysts for toluene degradation.

M. Zhang *et al.* utilized a hydrothermal technique to synthesize In_2_S_3_ in a nanoscale form. This nanomaterial was then employed to fabricate a composite photocatalyst consisting of In_2_S_3_ and g-C_3_N_4_. The process of toluene photocatalytic decomposition was investigated, and a feasible mechanism was proposed. The In_2_S_3_/g-C_3_N_4_ heterojunctions exhibited the highest photocatalytic degradation when a 40% loading of In_2_S_3_ was used.^184^ B. N. R. Winayu *et al.* enhanced the TiO_2_ catalyst by introducing sulfur and nitrogen (S, N) components and reduced graphene oxide (rGO) through doping. The most efficient photocatalytic degradation of toluene was achieved using a combination of 1 wt% reduced graphene oxide (rGO) and 0.05 wt% nitrogen-doped titanium dioxide (N_0.1_TiO_2_).^185^ V. T. T. Ho *et al.* stated that the nanostructured Ir-doped TiO_2_ is a highly effective photocatalyst that produces a superb material for reducing the risk of gaseous toluene. The material had a large surface area and had a consistently spherical shape of 10–15 nm diameter.^186^ The composite of PIL (polyionic liquid)@TiO_2_ was formed using two different concentrations of polymerized ionic liquid (low and high). The composite was then assessed for its ability to degrade toluene. The findings indicated that the PIL(low)@TiO_2_ composite exhibited higher activity compared to the PIL(high)@TiO_2_ composites.^187^ Z. Sun *et al.* synthesized a novel hierarchical heterostructured photocatalyst consisting of TiO_2_/Bi/Bi_2_MoO_6_ using a solvothermal technique. On the outermost layer of flower-like Bi_2_MoO_6_ nanospheres, the TiO_2_ nanoparticles were evenly dispersed. The results suggest that the combination of TiO_2_ can greatly improve the effectiveness of the photocatalytic oxidation of toluene using the hierarchical heterostructure TiO_2_/Bi/Bi_2_MoO_6_.^188^ Y. Bi *et al.* used zinc chloride (ZnCl_2_), zinc nitrate (Zn(NO_3_)_2_), and zinc acetate (Zn(CH_3_COO)_2_) to modify activated carbon fibers (ACFs). Subsequently, titanium dioxide (TiO_2_) was loaded onto the modified ACFs. The study found that the photocatalytic performance and adsorption of TiO_2_/ACF-Ac modified by Zn(CH_3_COO)_2_ were highest for the removal of toluene.^189^ The presence of a three-dimensional (3D) and directed structure enables efficient absorption of photons and rapid diffusion of volatile organic compounds (VOCs), surpassing the capabilities of catalysts in powder form. The researchers successfully created uniform and free-standing nanowire (NW) arrays of p-type Cu_2_O by subjecting Cu(OH)_2_ NWs to heat treatment. The Cu_2_O NWs, as they are created, exhibit exceptional performance in degrading 30 ppm toluene, with a degradation rate of 99.9% achieved within 120 minutes.^190^ P. Mohammadi *et al.* used a hydrothermal technique to deposit synthesized SrTiO_3_ onto graphene oxide (GO). Photocatalysts that were artificially created were utilized for the process of breaking down gaseous toluene dynamically using photocatalysis while being exposed to UV radiation.^191^ Rostami synthesized a TiO_2_ and bentonite photocatalyst by a method called co-precipitation and evaluated its catalytic efficiency in degrading *para*-nitrotoluene (PNT).^192^ Oxygen vacancies (OVs) can regulate photocatalytic activity by altering their electrical and/or band structures. A wide bandgap p-block metal combination containing OVs, indium oxyhydroxide (InOOH), produced using a one-pot hydrothermal approach, was used to investigate the effect of OVs on photocatalytic decomposition and toluene ring breakage. Validated modified InOOH improves photocatalytic potency by decreasing the energy limitation of critical intermediates for reaction during toluene degradation.^183^ X. Zhao *et al.* enhanced the performance of the C–USTiO_2_ photocatalyst by applying it to carbon cloth and conducted a study on its ability to continuously degrade toluene under LED light exposure. The results demonstrated that the removal of the degraded toluene can exceed 80% when a large concentration of CO_2_ is produced, and it exhibits exceptional cycle stability lasting for over 180 minutes.^193^ M. Wu *et al.* researched the use of CeO_2_ nanorods for the degradation of toluene using vacuum ultraviolet (VUV) catalytic oxidation. CeO_2_ nanorods were utilized in a system that involved VUV-photolysis, UV-PCO, OZCO, and UVOZCO processes. Utilizing VUV light instead of ozone catalytic oxidation can significantly enhance the efficiencies, increasing them from 12.9% to 83.2% when combined with the suggested catalyst.^194^ An efficient electrochemical method consisting of two steps was devised to produce a nanotube array of atomically dispersed Au-loaded WO_3_/TiO_2_ for the oxidation of volatile organic compounds (VOCs). The presence of vacancies (OVs) on the surface of WO_3_ greatly improved the separation and movement of photogenerated carriers, as well as the adsorption of toluene. This resulted in an 85.5% mineralization and 95.4% degradation rate for the removal of toluene.^195^ J. Lyu *et al.* fabricated a hollow heterophase junction by applying a layer of amorphous TiO_2_ onto anatase TiO_2_ hollow spheres. The findings demonstrated that the application of the amorphous TiO_2_ coating resulted in an augmentation of fine pores and intermediate pores in the photocatalyst, leading to an improved capacity for toluene adsorption.^196^ By adding nanodiamonds to ZnO, the photocorrosion problem can be solved for photocatalytic degradation of gaseous toluene. A table has been added showing the photocatalytic degradation of toluene Table 3 and Fig. 3 shows the process. Nanodiamond decoration resulted in lowered photoluminescence intensity and electrochemical impedance, enhancing ZnO light absorption, charge transfer, and photocatalytic toluene oxidation efficiency.^197^

**Table 3:** Data for the photocatalytic degradation of toluene using various catalysts

Toluene conc. (ppm)	Catalyst	Catalyst conc. (mg L^−1^)	Operational conditions	Lamp, power (W)	Degradation percentage (%), time (min)	Ref.
60	In_2_S_3_/g-C_3_N_4_	50	Visible light, RH 50–60%	Xe	89.7, 180	184
2	1 wt% rGO/S_0.05_N_0.1_TiO_2_	500	Visible light, RH 60%	Fluorescent, 10	72, 480	185
1900	Ir doped–TiO_2_	100	UV light, RH 70%	UV, 25	97, 8.5	186
50	PIL@TiO_2_/m-GO	1000	UV, RH 40%	UV, 8	97, 24	187
—	TiO_2_/Bi/Bi_2_MoO_6_	2000	UV	Xe, 320	26.08, 120	188
843	TiO_2_/ACF-Ac fabricated by Zn(CH_3_COO)_2_	151.2	UV, RH 40%	Xe, 300	70, 2400	189
30	Cu_2_O NWs	172.26	Visible, RH 74%	Xe, 300	99.9, 120	190
60	SrTiO_3_/rGO	400	UV light, RH 50%	UV, 8	98.65	191
50	TiO_2_/Bentonite	200	UV light	UV lamp	64, 120	192
50	InOOH	400	UV light	Xe, 300	75.8, 60	183
30	C–USTiO_2_	100	Visible light, RH 50%	LED, 1	80, 180	193
30	CeO_2_	1000	VUV light, RH 50%	VUV, 4	83.2, 144	194
300	WO_3_/TiO_2_	—	LED light	—	95.4, 30	195
23.6	THS@amorphous-TiO_2_	10	UV light	UV, 8	98.2, 240	196
50	TiO_2_/ND	100	UV	Xe, 50	100, 120	197
750	ZnAl_2_O_4_	—	UV	Black-light fluorescent lamp	90.25, 300	198
—	TiO_2_	—	UV, RH 35%	Germicidal lamp, 15	61.9, 180	199
160	TiO_2_	—	UV, RH 25–50%	Black lamp, 10	50–60, 5	200
400	TiO_2_	—	UV	Iron halogenide lamp, 500	52, 360	201
—	TiO_2_	—	UV	Mercury lamp, 300	90, 120	202

### Nitrobenzene

Since aromatic nitro compounds are frequently employed in industrial processes (such as the production of explosives, dyes and insecticides), they are present as contaminants in a variety of liquid sources, particularly surface water, and wastewater from industries.^203^ Since nitrobenzene (NB) is identified as a significant contaminant, it is selected as a model pollutant. It is an extremely hazardous material and the highest permitted level of NB is 1 mg L^−1^ in wastewater.^204,205^ Numerous factors, including the presence of anions, pH, light wavelength, and others, have an impact on nitrobenzene photocatalytic degradation utilizing UV radiation.^206^ The degradation working mechanism of nitrobenzene in the presence of several photocatalysts is described.^207,208^

22 Photocatalyst + *hν* (photon) → Photocatalyst (e_cb_^−^ + h_vb_^+^)

23 e_cb_^−^ + O_2_ → ˙O_2_^−^

24 Nitrobenzene → Catalyst surface

25 Nitrobenzene + ˙OH → Activated nitrobenzene

26 Activated nitrobenzene + ˙OH → Nitrophenol + Intermediate products

27 Nitrophenol + ˙OH → Degradation products

28 Degradation products + ˙OH → CO_2_ + H_2_O + Inorganic ions

**Fig. 4:**
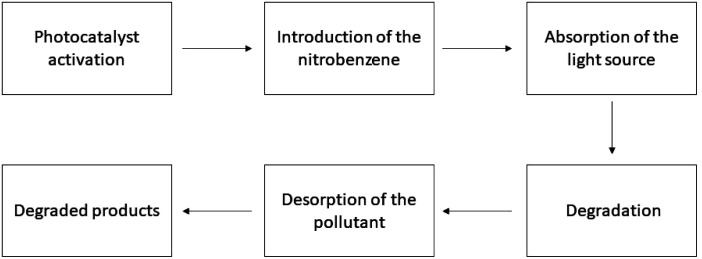
Working procedure of the photocatalysts for nitrobenzene degradation.

The study of the impacts of several factors, such as pH, anions, starting concentration, *etc.*, has been done because the rate of breakdown of nitrobenzene utilizing controlled UV radiation is quite significant when compared to that utilizing solar radiation, and a small amount of TiO_2_ (0.05%, w/v) was used.^209,210^ Degussa P-25 TiO_2_ was utilized as the photocatalyst in the majority of the nitrobenzene photocatalytic tests. Aldrich-TiO_2_ (pure anatase with a BET surface area of roughly 250 m^2^ g^−1^) was used in a few tests.^206^ Matthews *et al.* used immobilized TiO_2_ in a spiral-shaped reactor for the photocatalytic degradation of NB and other chemicals and accomplished around 95–100% degradation at the initial concentration between 1.75 and 4.25 mg L^−1^.^211^ Degussa P-25 was applied as the catalyst in photocatalytic degradation tests, and UV lamps with lights radiating at *λ*_max_ of 253 and 365 nm, respectively, were used. The two bulbs produced nearly identical deterioration.^212^ When it comes to 4-chlorophenol degradation, it has been discovered that utilizing pulsed photocatalysis makes little distinction in terms of TOC elimination at shorter and longer wavelengths. It should be mentioned that 387 nm is the *λ*_min_ for anatase TiO_2_.^213^ The pH has an impact on the ionizable organic molecules' photocatalytic breakdown. The significance of pH on the photocatalytic destruction of NB was assessed within a pH value range of 4–10, in a solution containing 2.52 × 10^−4^ M of pollutants. The ideal photocatalyst concentration was determined to be 0.5 wt% Fe–TiO_2_ = 250 mg L^−1^, with an irradiation period of 60–240 minutes.^214^ A table has been added showing the photocatalytic degradation of nitrobenzene Table 4 and Fig. 4 shows the process. It has been discovered that, given the specified conditions, pH 7 is ideal for NB photocatalytic breakdown.^205^

**Table 4:** Data for the catalytic degradation of nitrobenzene using various catalysts

Nitrobenzene conc. (mg L^−1^)	Catalyst	Catalyst conc. (mg L^−1^)	Operational conditions	Lamp, power (W)	Degradation percentage (%), time (min)	Ref.
50	SrFeO_3−*δ*_	0.001	UV	Mercury vapour, 125	99%, 360	215
50	P-25	—	UV	125	95%, 480	216
40	AuNPs/HPW/TiO_2_-NTs	—	Visible light	Low-pressure mercury vapor lamp, 15	90%, 30	217
—	Ag/ZnO nanoflowers	—	UV	Tungsten lamp, 60	98%, 100	218
61.5	TiO_2_	—	Visible light	125	58.46%, 210	219
—	TiO_2_/g-C_3_N_4_/G	7.5	UV	Xenon lamp, 300	97%, 240	220
25	H_3_PW_12_O_40_ supported on TiO_2_	10	Visible light	Tungsten light, 500	88%, 390	221
50		—	UV	Mercury vapor lamp, 200	96%, 240	222
50	TiO_2_-POMs	—	UV	—	86.4%, 180	223
1900	TiO_2_-SA-Arg particles	—	UV	UV lamp	93.7%, 120	224

### Cyclohexane

A common volatile organic compound (VOC) that presents significant dangers to both humans and the environment is cyclohexane.^225^ An extremely significant industrial procedure is the breakdown of cyclohexane to produce cyclohexanol and cyclohexanone which are utilized globally as chemical precursors for the synthesis of caprolactam and adipic acid.^226,227^ Photocatalytic techniques for the degradation of cyclohexane in both solid heterogeneous and homogeneous stages have received a lot of research attention in recent years.^228^ In heterogeneous environments, semiconductors along with oxides are being used as photocatalysts to oxidize cyclohexane. A number of semiconductors have been used, including CeO_2_, WO_3_, Sn/Sb, ZrO_2_, ZnO, V_2_O_5_, SnO_2_, Sb_2_O_4_ and mixed oxides.^229^ In the presence of various types of photocatalysts, cyclohexane degrades *via* the following mechanism.^230,231^

29 Photocatalyst + *hν* (photon) → Photocatalyst (e_cb_^−^ + h_vb_^+^)

30 h_vb_^+^ + H_2_O → ˙OH + H^+^

31 h_vb_^+^ + OH^−^ → ˙OH

32 Cyclohexane + ˙OH → Intermediate products

33 Intermediate products + ˙OH → Further degraded products

34 Intermediate products + ˙OH/O_2_ → CO_2_ + H_2_O + Inorganic ions

**Fig. 5:**
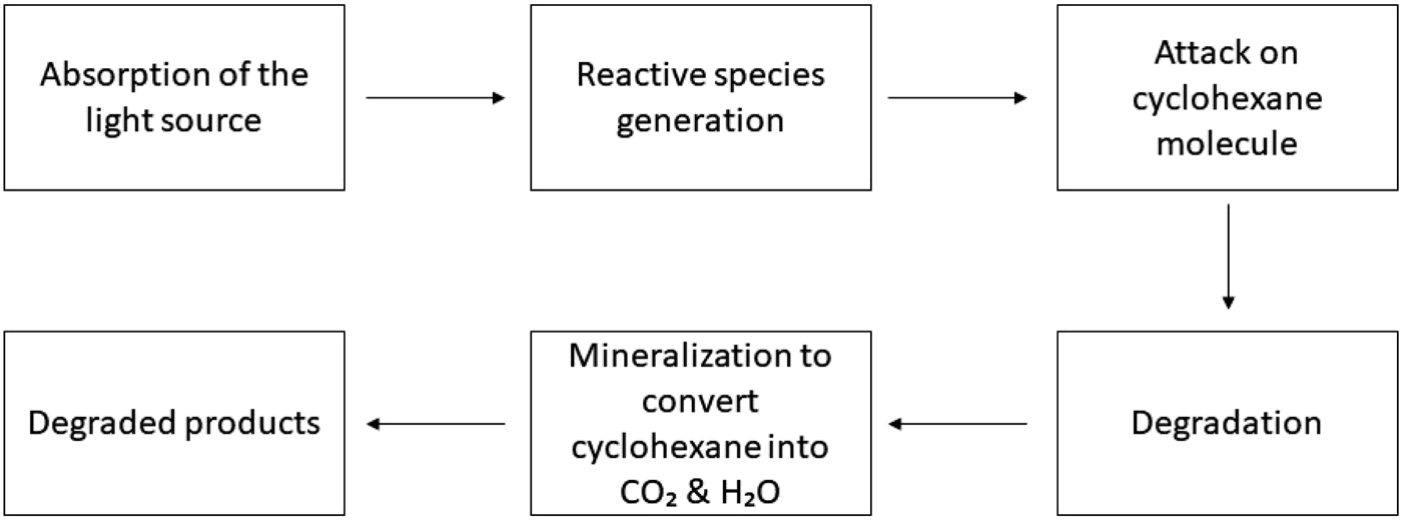
Working procedure of the photocatalysts for cyclohexane degradation.

Xiao *et al.* discussed the photocatalytic characteristics of silver nanoparticles loaded on the nanocrystals of tungsten oxide when cyclohexane was being photo-catalytically degraded.^232^ In standard manufacturing processes, cyclohexane is degraded at 150 °C using a homogeneous cobalt-based catalyst.^228^ Variations in the emitted photon flux and the irradiation wavelength during continuous irradiation result in notable variations in substance outputs and selectivity values during the photocatalytic degradation of cyclohexane by the help of TiO_2_ in a pure liquid organic phase.^233^ The photodegradation of cyclohexane proceeded with hydrogen peroxide at ambient temperature, assisted by a copper(ii)-exchanged Y zeolite (CuY). A table has been added showing the photocatalytic degradation of cyclohexane Table 5 and Fig. 5 shows the process. Following 6 hours of processing, cyclohexanol and cyclohexyl hydroperoxide with 37% and 54% selectivities, respectively, were obtained as the major products.^247^

**Table 5:** Data for the catalytic degradation of cyclohexane using various catalysts

Cyclohexane conc. (mg L^−1^)	Catalyst	Catalyst conc. (mg L^−1^)	Operational conditions	Lamp, power (W)	Degradation percentage (%), time (min)	Ref.
200	Pt/TiO_2_	—	UV, temp: 100 °C with 0.5% Pt loading	Osram Ultra-Vitalux lamp, 300	Close to 100%, —	234
—	Ti^3+^ self-doped TiO_2_	—	Visible light, temp: 40 °C	Xenon lamp	95%, 420	235
—	WO_3_/Co–Pt	100	UV	—	93%, 720	236
—	Degussa P-25	0.001	Visible light	Metal halide lamp	Around 40%, 180	237
—	WO_3_–TiO_2_ mixed catalysts	50	Visible light	Xenon lamp, 500	97%, 60	238
—	Au/TiO_2_	0.001	Visible light, temp: 25 °C	Mercury lamp, 50	50%, —	239
—	Degussa P25	0.001	UV, temp: 650 °C	Xenon lamp, 450	—, 60	240
523	TiO_2_	—	UV, temp: 30 °C	Black light lamp, 20	63%, 5	241
—	TiO_2_	0.001	UV, —	Mercury lamp, 50	—, 10	239
—	Ag-substituted and impregnated nano-TiO_2_	0.001	UV, temp: below 35 °C	A high-pressure mercury vapor lamp, 80	Around 10%, 6	242
—	TiO_2_	—	UV, temp: 140–180 °C	A high-pressure mercury lamp, 100	Over 90%, —	243
—	TiO_2_	0.001	UV, temp: 60 °C	Medium pressure mercury-vapor lamp, 450	Over 95%, —	244
—	Na_4_W_10_O_32_	0.05	UV, —	Medium pressure mercury-arc lamp, 125	—, 3	245
—	Fe-modified C-doped Cr_2_O_3_	—	Visible light, temp: 25 °C	Xenon lamp, 300	—, 5	246

### Refinery oil

Several methods may be used for the treatment of oil refinery effluents which include adsorption, Fenton oxidation, electro-floatation–coagulation, photocatalytic degradation/oxidation, chemical flocculation–coagulation, and membrane filtration.^248–253^ These procedures either produce insignificant impurities or need prolonged durations to eradicate the impurities.^254–256^ Conventional methods like adsorption or membrane separation produce an inferior contaminant by moving the contamination from one phase to another, and the reusability of adsorbents is uncertain.^257,258^ Bacterial degradation requires a significant amount of time to break down pollutants and is not suitable for the majority of organic compounds found in oil refinery wastewater.^259–261^ Photocatalytic degradation techniques have attracted significant attention due to their ability to break down a wide range of organic compounds utilizing suitable photocatalysts.^52,262,263^ The degradation of pollutant chemicals is caused by the hydroxyl radical (OH), which can react with organic compounds and break them down and degrade them.^264,265^ The mechanism for refinery oil degradation in the presence of various photocatalysts is given.^266,267^

35 Photocatalyst + *hν* (photon) → Photocatalyst (e_cb_^−^ + h_vb_^+^)

36 e_cb_^−^ + O_2_ → ˙O_2_^−^

37 h_vb_^+^ + H_2_O → ˙OH + H^+^

38 h_vb_^+^ + OH^−^ → ˙OH

39 Oil → Catalyst surface

40 Oil + ˙OH → Degradation products

41 Oil + ˙O_2_^−^ → Degradation products

42 Degradation products + ˙OH → CO_2_ + H_2_O + Inorganic ions

**Fig. 6:**
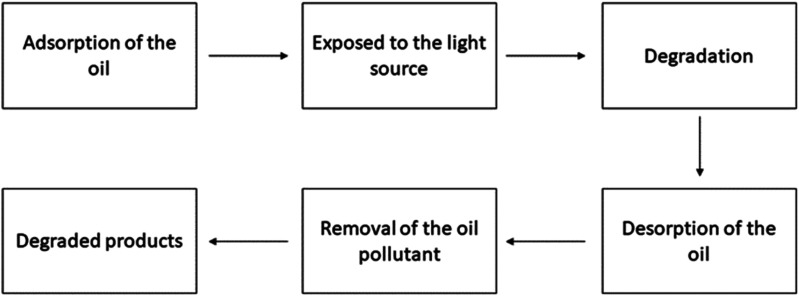
Working procedure of the photocatalysts for refinery oil degradation.

B. Ogoh-Orch *et al.* studied BiOI-sensitized TiO_2_ (BiOI/TiO_2_) nanocomposites with varying amounts of BiOI deposited *via* sequential ionic layer adsorption and reaction (SILAR) and found that they perform well in water under visible (>400 nm) irradiation for crude oil degradation. The BiOI/TiO_2_ heterojunction separates photogenerated charges, improving degradation efficiency.^268^ Actual wastewater from a refinery, containing a variety of aromatic and aliphatic organic compounds, was treated using nanoparticles (specifically TiO_2_ and ZnO). The degradation ability of the organic contaminants was reduced from 98.57% to 89.482% when the photocatalysts changed from TiO_2_ to ZnO.^267^ D. A. Aljuboury *et al.* investigated the application of ZnO/TiO_2_/H_2_O_2_ using visible light (1000 W m^−2^), to decrease the total organic carbon (TOC) content in the actual petroleum wastewater obtained from Sohar Refinery Company (SRC). The treatment efficiency for total organic carbon (TOC) at pH 5.5 increased significantly compared to that of the TiO_2_ procedure.^269^ Z. Ghasemi *et al.* examined the photocatalytic oxidation of organic contaminants in petroleum refinery wastewater (PRWW) utilizing synthesized nano-TiO_2_ incorporated into Fe-ZSM-5 zeolite and UV light. Results indicate optimal photodegradation efficiency at 3 g L^−1^ photocatalyst concentration, pH 4, 45 °C temperature, and 120 min UV irradiation.^270^ Shahrezaei investigated TiO_2_ photocatalysis for the primary degradation of phenol and phenolic compounds in refinery wastewater. Under optimal conditions, 90% phenol removal was achieved in 2 hours.^271^ The user created a composite membrane by combining polyvinylidene and titanium dioxide (PVDF/TiO_2_) and then treated it using the hot-pressing method. A table has been added showing the photocatalytic degradation of cyclohexane Table 6 and Fig. 6 shows the process. This treatment was done to increase the bonding between the TiO_2_ and the membrane surfaces, to employ the membrane to degrade oil in wastewater.

**Table 6:** Data for the photocatalytic degradation of refinery oil using various catalysts

Target compound	Compound conc. (ppm)	Photocatalyst	Catalyst conc. (mg L^−1^)	Operation conditions	Lamp, power (W)	Degradation percentage (%), time (min)	Ref.
Crude oil	200	BiOI/TiO_2_	—	Visible light	LED, 13	85.62, 180	268
Refinery oil	99.64	TiO_2_	100	UV light, pH 6	UV, 11	98.57, 120	267
Refinery oil	99.64	ZnO	100	UV light, pH 3	UV, 11	89.48, 120	267
Oil in petroleum wastewater	15–22	TiO_2_/ZnO/H_2_O_2_	H_2_O_2_ = 850	Visible light, pH 5.5	LED, 1000	37, —	269
			ZnO = 590				
			TiO_2_ = 700				
Petroleum refinery wastewater	500	TiO_2_/Fe-ZSM-5	3000	UV light, pH 4	UV, 8	66%, 120	270
Phenol	220	TiO_2_	100	UV light, pH 4	UV, 400	90%, 120	271
Phenol	70	Degussa P25	0.002–0.008	UV light, pH 5.5	Fluorescent T8 backlight blue bulb, 18	76%, 90	272
Soap oil & grease	480	Degussa P25	0.002–0.008	UV light, pH 5.5	Fluorescent T8 backlight blue bulb, 18	88%, 90	272
Refinery oil	—	TiO_2_	100	UV light, pH 3	UV, 400	93.92%, 60	273
Refinery oil	—	TiO_2_	0.0012	UV light, pH 4	UV, 11	40.68%, 120	274
Petroleum refinery wastewater	—	TiO_2_	100	UV light, pH 10	Mercury vapor, 6	TOC = 32% & TN = 67%, 90	275
Petroleum refinery wastewater	—	TiO_2_/ZnO/Degussa P25	0.0005–0.005	UV light, pH 3.5–9	Mercury vapor, 250	Phenols = 93%	276
						Dissolved organic carbon (DOC) = 63%	
						Oil and grease (OG) = over 50%, 60	
Petroleum refinery wastewater	400–600	TiO_2_	100	UV light, pH 3, temp: 45 °C	Mercury, 400	90%, 4	277
Petroleum refinery wastewater	Phenol = 0.002	TiO_2_	0.001	O_3_/UV, pH 7.16	Mercury, 100	Phenol = 99.9%	278
	COD = 1954					COD = 31.6%	
	Oil & grease = 298.8					Oil & grease = 5.2%	
	Sulfide = 13.3					Sulfide = 53%, 5	
Phenol	10	TiO_2_		UV, pH = 5		—, 6	279
Refinery oil	—	TiO_2_	—	UV light, pH 7–9	UV, 60	83%, —	280
Petroleum refinery wastewater	200–220	TiO_2_	100	UV light, pH 3, temp: 45 °C	Mercury, 400	78%, 120	281
Crude oil	0.005	TiO_2_ in zeolite	—	UV light, temp: 400 °C	Mercury, 150	Linear alkanes = 98.66%	282
						Branched alkanes = 97.31%	
						Cyclic alkanes = 96.04%	
						Aromatic compounds = 99.54%	
						Alkenes = 98.38%, 100	
Oil emulsion in distilled/sea water	25	Degussa P25	0.002	UV light, pH 4.5	Mercury, —	In distilled water 92% & in artificial seawater 43%, 3	283
Petroleum refinery wastewater	COD = 1326	Green nanocatalyst from the sepals of waste tomato	0.00025	UV light, —	UV, —	99.9%, 90	284
A synthetic oil–water emulsion	10 000	TiO_2_	0.002	UV light, —	T8 black light blue bulb, 18	68%, 30	285
Refinery wastewater	100	4-Chlorophenol	178.5	UV light, pH 5	Mercury lamp, 100	—, 80	286

## Effects of crystal size and surface area on photocatalytic degradation

Organic chemicals and the photocatalyst's surface coverage are directly correlated, and therefore surface morphology, such as crystal size and the surface area, must be taken into account during the photocatalytic degradation procedure.^287,288^ Since every chemical process occurs at the surface, the surface morphology of any photocatalyst is essential to its efficacy as a catalyst.^289^ The anatase phase with a range of 2.59 to 12.00 nm in TiO_2_ crystallite dimensions is visible in metal-doped TiO_2_ products. TiO_2_ has a specific surface area of between 100 and 500 m^2^ g^−1^.^290,291^ Sivalingam *et al.* used a solution combustion process where 8–10 nm pure anatase phase TiO_2_ with 156 m^2^ g^−1^ BET surface area was created. This TiO_2_ is commonly utilized for photocatalytic degradation of many dyes, including Orange G, Methylene Blue, Alizarin S, Methyl Red, and Congo Red. In this analysis, the crystal size of the photocatalyst was found to be 8 ± 2 nm.^292^ The photoactivity of the photocatalysts increased due to the higher surface area. It has been found that the photoactivity of the TiO_2_ while degrading the dye-like MB increased when the surface area of the catalyst increased from 63 m^2^ g^−1^ to 156 m^2^ g^−1^.^293^ For the maximum degradation of antibiotics like cefoxitin sodium, a novel BN/CdAl_2_O_4_ composite with a surface area of 14.34 m^2^ g^−1^ is used.^133^ Mushtaq *et al.* found a decrease in the degradation rate of the ofloxacin antibiotic due to the increase in the particle size and decrease in the surface area of the photocatalysts.^294^ The same scenario was also found during the advanced degradation of tetracycline antibiotics by graphene-ordered mesoporous silica.^295^ Zhou *et al.* used highly photoactive mesoporous anatase nanospheres that have a high specific surface area of 609 m^2^ g^−1^ for the degradation of toluene.^296^ The highest specific surface area (130.3 m^2^ g^−1^) of nano-sized TiO_2_ particles synthesized under ideal conditions is almost double that of Degussa P25 which is used for toluene degradation.^297^ R. J. Tayade *et al.* experimented with the degradation of nitrobenzene using nanocrystalline TiO_2_ of different surface areas, *i.e.* 259/199/166/124/91/2 m^2^ g^−1^.^216^ Photocatalytic oxidation of cyclohexane over TiO_2_ nanoparticles by molecular oxygen was carried out using different surface area photocatalysts ranging between 30 and 410 m^2^ g^−1^.^298^ TiO_2_ is made up of anatase and rutile with a mean particle size of 30 nm and a surface area of 50 m^2^ g^−1^ for the maximum degradation of refinery oil.^285^

## Mechanism of photocatalytic degradation

**Fig. 7:**
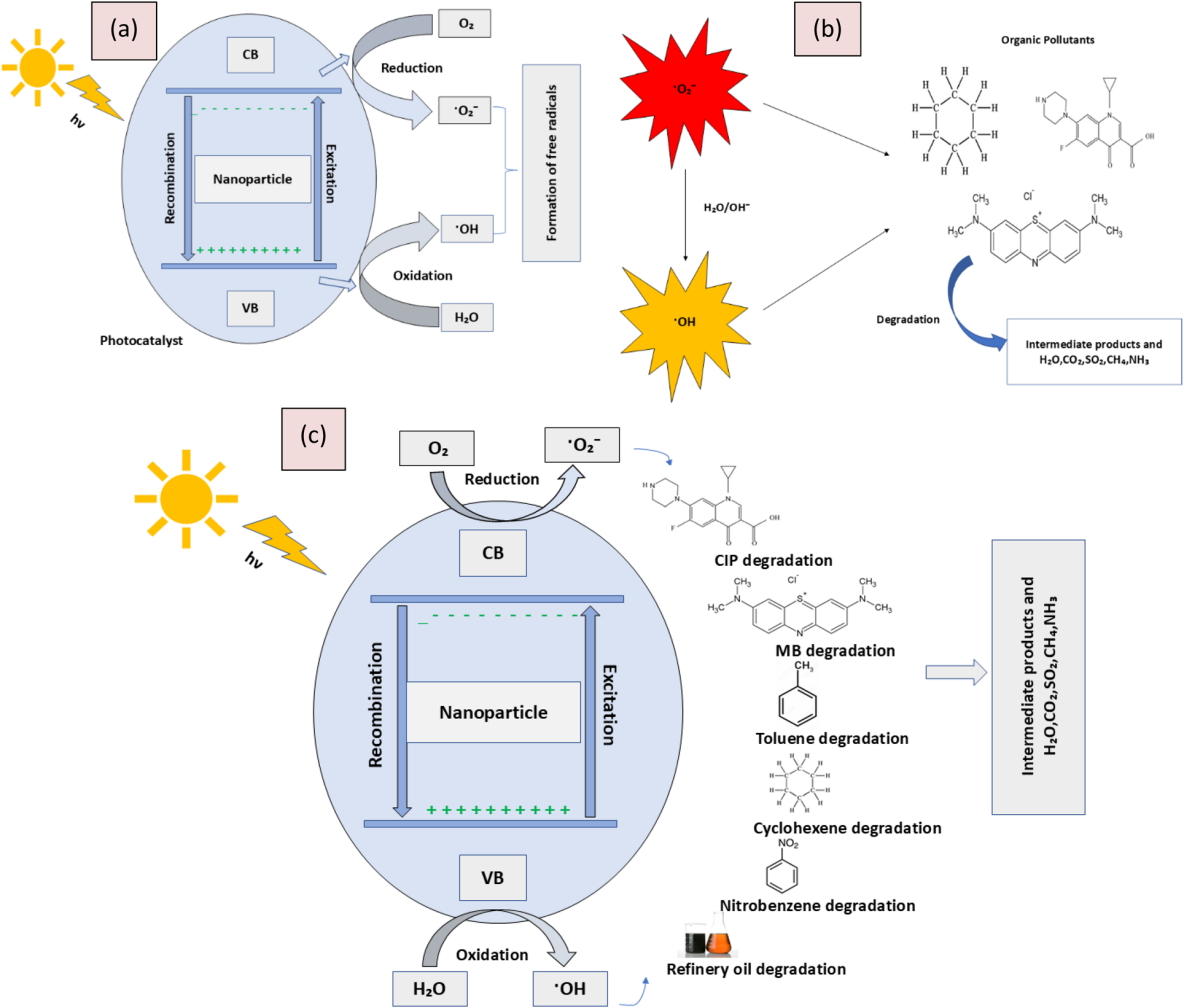
Illustration of (a) formation of free radicals, (b) degradation of the organic pollutants by radicals, and (c) overall photocatalytic degradation mechanism.

Photocatalytic degradation is a process where light energy, typically from UV or visible light, activates a photocatalyst, such as titanium dioxide (TiO_2_). When the photocatalyst absorbs light, it generates electron–hole pairs. These electron–hole pairs can initiate redox reactions that produce reactive oxygen species (ROS) like hydroxyl radicals and superoxide anions. These ROS are highly reactive and can break down organic pollutants, converting them into less harmful substances like water, carbon dioxide, and inorganic ions. The overall mechanism involves light absorption, generation of electron–hole pairs, formation of ROS, and degradation of pollutants (Fig. 7).

## Conclusion

Various photocatalysts are used depending on the variation in organic pollutants. Titanium dioxide (TiO_2_) is the most broadly applied photocatalyst, known for its maximum ability, stability, and non-toxicity. It is primarily activated by UV light. Zinc oxide (ZnO) is another effective photocatalyst with properties similar to those of TiO_2_ but with some advantages under certain conditions. Recent research includes materials like cadmium sulfide (CdS), tungsten oxide (WO_3_), and various metal–organic frameworks (MOFs) as effective photocatalysts. Scientists are working on photocatalysts that are triggered by visible light in order to improve the process's applicability and reduce energy consumption in the real world. This review scrutinizes the variance in the degradation rate of organic pollutants under different conditions such as different pH levels, different concentration levels, various composites of the photocatalysts, different surface areas and sizes of the photocatalysts, and so on. This review will help to identify the optimum parameters for the maximum amount of organic pollutant degradation. The goal of this field's ongoing research and development is to broaden the use of catalytic technologies and overcome current obstacles to ensure cleaner soil and water thus leading to a more sustainable environment. Greater prospects for the use of photocatalysis in the destruction of dangerous organic pollutants may arise from a greater understanding of the process and its operating parameters.

## Author contributions

Abdullah Al Miad and Shassatha Paul Saikat collected the data and wrote the draft and original manuscript. Md. Sahadat Hossain conceived and designed the review, analyzed the data, and assisted in writing the manuscript. Md. Kawcher Alam assisted in collecting data. Newaz Mohammed Bahadur and Samina Ahmed supervised the findings of this work. Samina Ahmed supervised the overall work and managed the required facilities.

## Conflicts of interest

There are no conflicts to declare.

## Data Availability

Data will be made available on request.
